# Comparative efficacy of Clinpro^TM^ clear fluoride treatment and Duraphat^TM^ varnish as remineralizing agents for white spot lesions around orthodontic brackets: an *in vitro* study

**DOI:** 10.3389/froh.2026.1926039

**Published:** 2026-09-24

**Authors:** Sukhontamala Phuanglerngsri, Aggasit Manosudprasit, Apa Juntavee, Araya Pisek

**Affiliations:** 1 Division of Orthodontics, Department of Preventive Dentistry, Faculty of Dentistry, Khon Kaen University, Khon Kaen, Thailand; 2 Division of Pediatrics, Department of Preventive Dentistry, Faculty of Dentistry, Khon Kaen University, Khon Kaen, Thailand; 3 Division of Dental Public Health, Department of Preventive Dentistry, Faculty of Dentistry, Khon Kaen University, Khon Kaen, Thailand

**Keywords:** clinpro clear, duraphat, fluoride varnish, *in vitro*, microhardness, orthodontic brackets, remineralization, white spot lesions

## Abstract

**Introduction:**

Fixed orthodontic appliances increase plaque accumulation and the risk of enamel white spot lesions (WSLs). Although Duraphat™ Varnish (22,600 ppm fluoride) is widely used for WSLs management, limited evidence exists regarding the remineralization efficacy of the newer Clinpro™ Clear Fluoride Treatment (9,500 ppm fluoride).

**Objective:**

To compare the remineralization efficacy of Duraphat™ Varnish, single-application Clinpro™ Clear, double-application Clinpro™ Clear, and a no-treatment control on artificial WSLs adjacent to orthodontic brackets.

**Methods:**

Sixty-eight extracted human premolars were randomly allocated to four groups (*n* = 17 each). Following artificial lesion formation, specimens received the assigned fluoride treatment and underwent a 7-day pH-cycling regimen. Remineralization was assessed using Vickers surface microhardness, polarized light microscope (PLM), scanning electron microscope (SEM), and energy-dispersive x-ray spectroscopy (EDS). Data were analyzed using Welch ANOVA and the Games-Howell *post hoc* test (α = 0.05).

**Results:**

Duraphat™ Varnish demonstrated significantly greater hardness recovery than single-application Clinpro™ Clear (*p* < 0.001) and the no-treatment control (*p* < 0.001). Double-application Clinpro™ Clear produced significantly greater remineralization than a single application (*p* < 0.05) and achieved outcomes approaching those of Duraphat™ Varnish (*p* < 0.05). These findings were supported by percentage hardness recovery and remineralization potential. Qualitative PLM, SEM, and EDS analyses consistently demonstrated greater remineralization in all fluoride-treated groups than in the untreated control.

**Conclusion:**

Under the present *in vitro* conditions, Duraphat™ Varnish produced the greatest remineralization, while a double application of Clinpro™ Clear significantly enhanced its remineralization efficacy compared with a single application. These findings suggest that repeated application may improve the performance of Clinpro™ Clear; however, further *in vivo* and clinical studies are required to confirm its clinical effectiveness.

## 1 Introduction

White spot lesions (WSLs) represent a prevalent and clinically significant complication associated with fixed orthodontic therapy. These lesions arise as a consequence of enamel demineralization driven by organic acids generated through the metabolic activity of cariogenic bacteria residing within dental plaque. The presence of orthodontic brackets, bands, and auxiliary appliance components substantially compromises oral hygiene efficacy, thereby creating conditions conducive to plaque accumulation at the enamel surface. Sustained plaque retention in an acidic oral environment disrupts the dynamic equilibrium between demineralization and remineralization, resulting in net subsurface mineral loss. This process manifests clinically as the characteristic opaque white discoloration of the enamel surface that clinically defines WSLs (1–3)

The prevalence of WSLs among orthodontic patients is high, ranging from 45.8% to over 90% (4–6). The risk is greatest during the first six months of treatment, when lesion development is most rapid, and continues to increase at a slower pace through 12 months (7). Beyond their effect on appearance, WSLs can progress to actual cavities if left untreated, adding to the overall cost and complexity of dental care (8–10).

Fluoride application is considered the standard approach for both preventing and treating WSLs (11, 12). Topical fluoride supports remineralization by encouraging the formation of fluorapatite and fluorohydroxyapatite, which resist acid attack more effectively than natural hydroxyapatite (13–16). Among the fluoride products currently available, Duraphat™ Varnish (5% sodium fluoride), containing 22,600 ppm fluoride, is one of the most widely used in-office agents. Its rosin-based formula exhibits good adhesion to the tooth surface, with the film remaining on the enamel for a prolonged period, allowing slow fluoride release and a high fluoride reservoir. However, a limitation of the rosin-based formulation is that it sets quickly upon contact with saliva. Additionally, its yellowish-brown color and thick texture may be less well accepted by patients (17). A newer product, Clinpro™ Clear Fluoride Treatment (2.1% sodium fluoride), containing 9,500 ppm fluoride, has not yet been evaluated in published research regarding its properties. The manufacturer states that this product offers two key advantages: a clear appearance and a water-based gel formulation. This formulation dissolves and disperses more quickly than alternatives, allowing for faster fluoride release, which provides a convenient contact time for clinical use. However, this faster dissolution may also reduce contact time with the tooth surface, potentially causing more rapid depletion and a smaller overall fluoride reservoir.

While several studies have examined the relative effectiveness of different fluoride products under the specific conditions created by orthodontic brackets (18, 19), a direct comparison between Clinpro™ Clear Fluoride Treatment and Duraphat™ Varnish has not yet been made. No published studies confirm whether these two products achieve comparable remineralization outcomes on enamel surfaces around orthodontic brackets (20). Therefore, the effectiveness of Clinpro™ Clear Fluoride Treatment in this clinical context remains to be established.

The present study aimed to evaluate and compare the remineralization ability of Duraphat™ Varnish with Clinpro™ Clear Fluoride Treatment (single and double applications) against a non-treatment control on artificially induced WSLs on enamel surfaces with bonded brackets in the laboratory, using a 7-days pH cycling model.

### 1.1 Research hypothesis

H_0_: There is no significant difference in remineralization potential among Duraphat Varnish and Clinpro Clear fluoride treatment and non-treatment group.
H_A_: There is a significant difference in remineralization potential between Duraphat Varnish and Clinpro Clear fluoride treatment and non-treatment group.

### 1.2 Research objectives

To quantify and compare the remineralization efficacy of Duraphat™ Varnish, single- and double-application Clinpro™ Clear Fluoride Treatment, and a non-treatment control on artificial white spot lesions around orthodontic brackets, using Vickers surface microhardness as the primary quantitative outcome.

## 2 Materials and methods

### 2.1 Experimental design and sample preparation

This *in vitro* experimental study used a pH cycling model to evaluate the remineralization ability of fluoride agents on WSLs around orthodontic brackets **(**Figure 1**)**. Ethical exemption was obtained from the Khon Kaen University Ethics Committee for Human Research (KKUEC; No. HE682277) before the study began.

**Figure 1:**
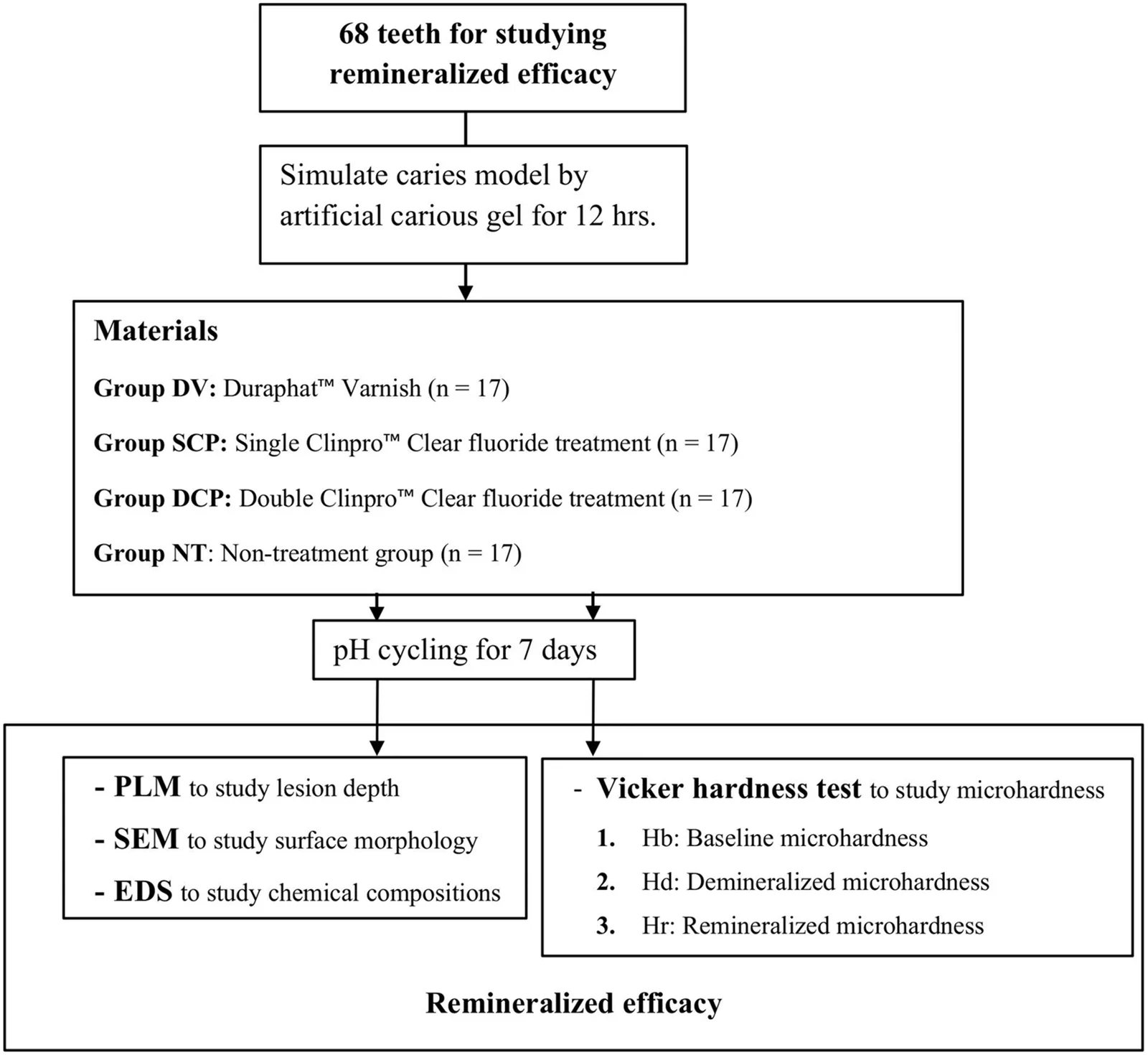
Flow chart of the experimental process.

Sample size was calculated using G*Power 3.1.9.4 software based on a previous study by Iamviteevanitch et al. (21), using Welch ANOVA parameters (effect size f = 0.52, α = 0.05, powe*r* = 90%). The minimum estimated sample size per group was 14 teeth; therefore, 17 teeth per group were prepared.

Total sixty-eight sound human premolars were initially collected from the Faculty of Dentistry, Khon Kaen University, and stored in 0.1% thymol solution until being used within 3 months of extraction. Inclusion criteria were sound, unrestored premolars extracted for orthodontic, functional, or periodontal reasons. Exclusion criteria included teeth with dental caries, fluorosis, enamel hypoplasia, existing white or brown spot lesions, or teeth that were impacted.

Included teeth were cleaned with pumice, mounted in a 5 mL disposable PVC syringe using epoxy resin, and buccal sections were obtained using a precision sectioning machine (Mecatome T180, Presi, France) under continuous water cooling. Specimens were progressively polished using silicon carbide sandpaper (P1000, P1500, P2000, P4000), and final polishing was carried out at 60 rpm for 1 min (Nano 1000s, Pace Technologies, Canada). A 4 × 5 mm enamel window was marked by applying acid-resistant nail varnish (Revlon, USA) to all surrounding surfaces **(**Figure 2**)** (21).

**Figure 2:**
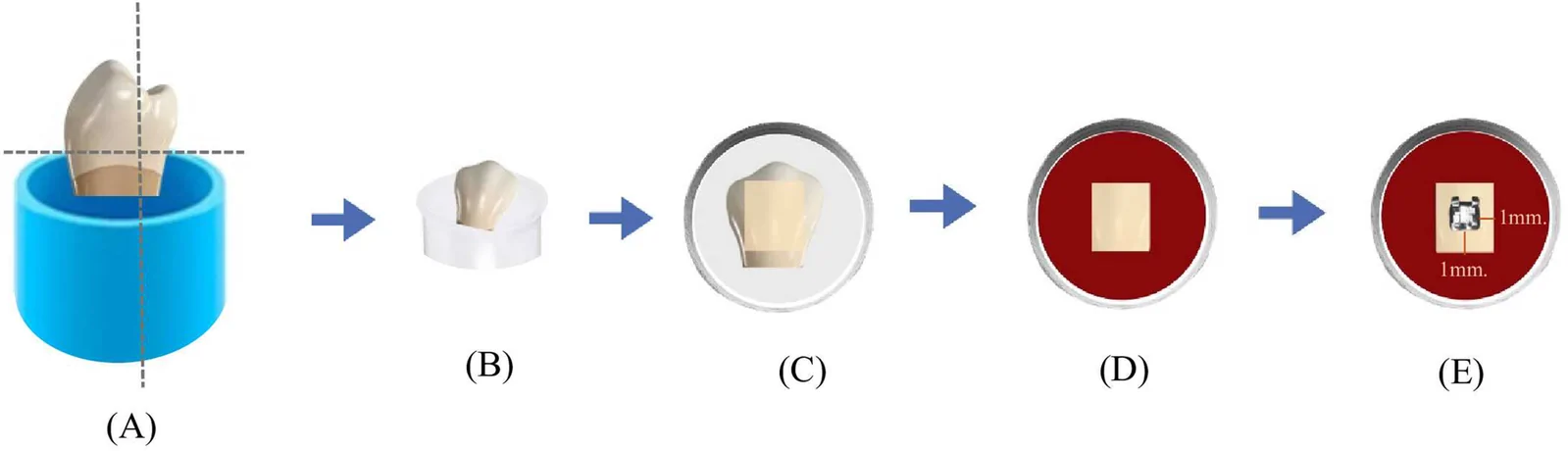
Specimen preparation and enamel surface preparation: **(A)** extracted premolars were mounted on PVC tube and separated from central groove. **(B)** Mounting the buccal surface facing the enamel with resin on a disposable syringe 5 mL. **(C)** Polishing the buccal surface with sandpaper. **(D)** The samples were coated with acid-resistant nail varnish, leaving exposed a 4 × 5 mm^2^ window. **(E)** After acid challenge lower anterior bracket were placed in the middle of the window.

### 2.2 Artificial carious induction

Artificial WSLs were created by placing specimens in a acid gel for 12 h at 37 °C following a modified Silverstone method (22). Specimens were washed thoroughly with DI. This process was expected to produce lesions approximately 100 µm deep, which corresponds to roughly three months of natural demineralization (23).

### 2.3 Bracket placement

After artificial WSLs induction, a 0.022-inch MBT metal bracket for lower anterior teeth 2 × 3 mm (W&H Tech, Thailand) was bonded to the center of the window using Transbond XT™ light cure primer and adhesive system (Solventum, Minnesota, USA) in a standard area of 1 × 1 mm at the bracket base, followed by light curing and removing excess adhesive.

### 2.4 Vickers microhardness testing

Surface microhardness was measured using a Vickers microhardness tester (Future Tech FM, Japan) with a 50 g load applied for 15 s. The brackets were removed before measurement, and the Vickers tester was calibrated beforehand. Indentations were made at 500 µm intervals from the occlusal, cervical, mesial, and distal margins of the bracket. Diagonal lengths were measured, and Vickers hardness number (VHN) values were calculated using FT ARS software v1.15.13 (Future Tech FM, Japan) **(**Figure 3**)**. Surface microhardness were measured at three time points: baseline surface microhardness (Hb) prior to demineralization, demineralized surface microhardness (Hd) following 12 h of acid gel exposure, and remineralized surface microhardness (Hr) after remineralization treatment combined with pH cycling. The measurement was repeated four times for each timepoint (occlusal, cervical, mesial, and distal areas). These values were used to calculate percentage hardness recovery (%HR) (24)

$$\%HR=\frac{Hr-Hd}{Hb-Hd}\;\times \;100$$

and percentage remineralization potential (%RP) (24).

$$\%RP=\frac{Hr-Hr_{of\;NT\;group}}{Hd}\;\times \;100$$

**Figure 3:**
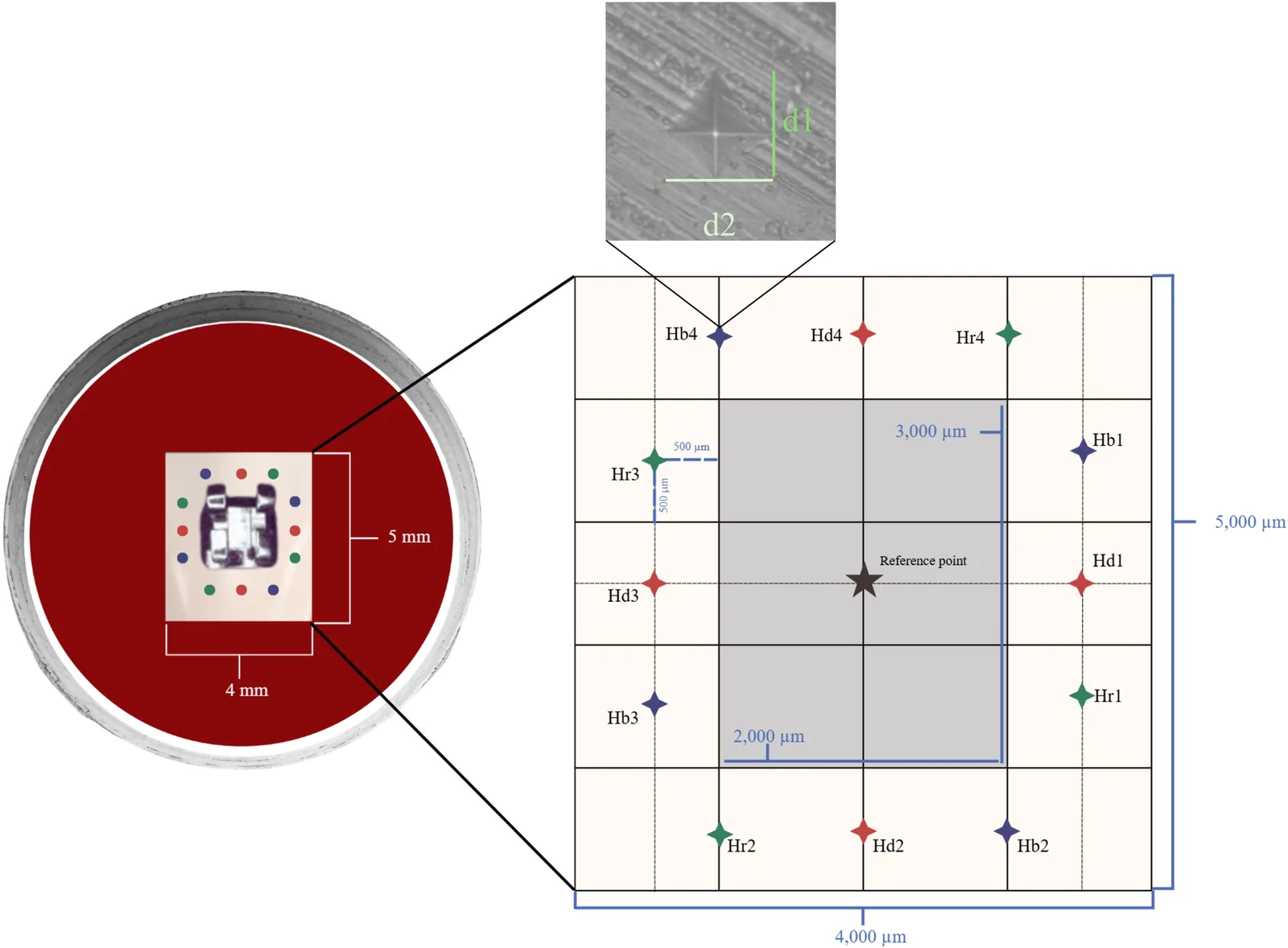
Vickers microhardness measurement: the indentation was measured for diagonal lengths (D1 and D2) and the Vickers hardness number was calculated. Hb: baseline surface hardness; Hd: demineralized surface hardness; Hr: remineralized surface hardness.

After demineralization, specimens were ranked by Hd values and assigned by stratified randomization into four equal groups (*n* = 17 each) to reduce operator bias, bracket removal and the final VHN measurement were carried out with the operator unaware of group assignment.

### 2.5 Fluoride application protocols

Duraphat™ Varnish (DV): 0.0025 g applied per surface in a circular motion around the bracket, left in place for the duration specified by clinical practice guidelines (25–28), then removed with DI water—moistened cotton swab before pH cycling.

Single application of Clinpro™ Clear fluoride treatment (SCP):0.0025 g applied in a circular motion around the bracket, left for 15 min as manufacturer instruction (20), then rinsed with DI water before pH cycling.

Double application of Clinpro™ Clear fluoride treatment (DCP): The same procedure as SCP (20) was performed before pH cycling, with a second identical application carried out at 84 h of pH cycling.

Non-treatment control group (NT):The specimens were stored in DI water (29) during the treatment period and went through pH cycling without any fluoride application.

### 2.6 The pH cycling protocol

All specimens underwent a standard 7-day pH cycling process at 37 °C with continuous movement at 50 rpm (WiseBath, Daihan Science, South Korea), simulating the changing oral environment. Each daily cycle included 12 h in DI water, 1 h 2 times in DS, and 5 h 2 times in RS (24), daily. Solutions were freshly prepared for each cycle. Specimens were stored individually in 24 well plates with 2 mL of the relevant solution **(**Table 1**)** and **(**Figure 4**)**.

**Table 1:** Composition of the experimental solutions used throughout the study.

Solution	Composition	Purpose
Acid gel	0.1 mol/L lactic acid 500 mg/L hydroxyapatite 20 g/L Carbopol™ (adjusted pH to 5.0 by 1 mol/L sodium hydroxide)	Artificial lesion formation
Remineralizing solution	1.5 mM Calcium chloride 0.9 mM Sodium dihydrogenphosphate 0.15 M Potassium chloride (adjusted pH to 7.0 by 1 molar sodium hydroxide)	pH cycling
Demineralizing solution	2.2 mM Calcium chloride 2.2 mM Potassium hydrogenphosphate 0.05 M Acetic acid (adjusted pH to 4.4 by 1 molar Potassium hydroxide)	pH cycling
Deionized water	Deionized water	Storage and washing

**Figure 4:**
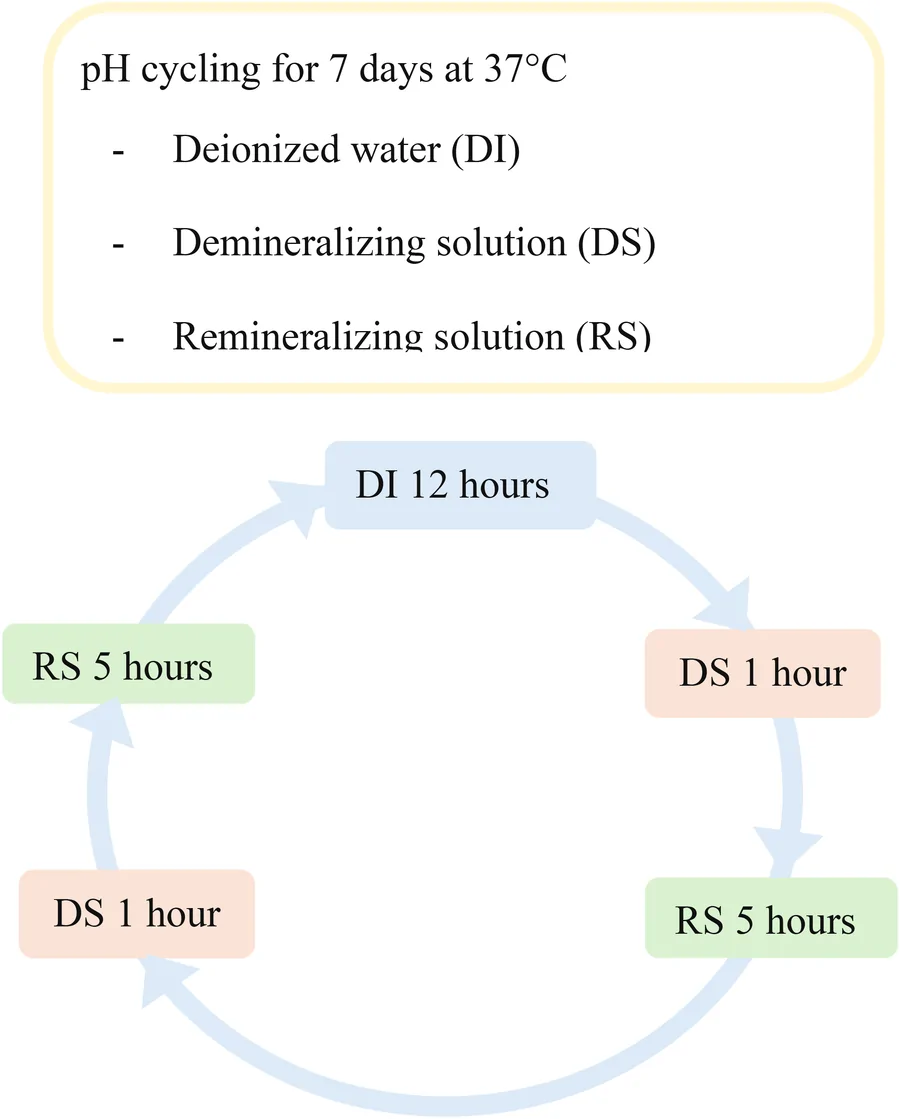
The pH cycling process.

### 2.7 Polarized light microscope (PLM)

The PLM was calibrated according to the manufacturer's recommendations before image acquisition. A subset of 12 specimens (3 from each treatment group) were cut lengthwise to a thickness of 150 to 200 µm (30) and examined under a PLM (Eclipse 80i, Nikon, Japan) at 20  ×   magnification. The dark zone that indicates the demineralized area (positive birefringence) was used to assess lesion depth and remineralization in a descriptive manner.

### 2.8 Scanning electron microscopy (SEM) and energy dispersive x-ray spectroscopy (EDS)

The SEM/EDS system was routinely maintained and calibrated according to the manufacturer's recommendations by the central instrumentation facility prior to specimen analysis. After air drying for 24 h, a subset of 12 specimens (3 from each treatment group) was sputter-coated with gold (K550X Emitech, England) for 3 min and stored in a desiccator. SEM analysis was performed at 2,000  ×  magnification (Hitachi S3000N, Japan). EDS was carried out under high vacuum conditions (5–15 kV accelerating voltage, 7.2–14.2 mm working distance) to determine weight percentages of Ca, P, F, O, C, and the Ca/P ratio (31).

### 2.9 Statistical analysis

Data were analyzed using SPSS v20 (IBM, USA). Normality was assessed with the Shapiro Wilk test. Group comparisons of HR, %HR, and %RP were performed using Welch ANOVA with Games Howell *post hoc* tests (significance level: *p* ≤ 0.05). Results are reported as means ± standard deviations. PLM, SEM, and EDS findings were described qualitatively.

## 3 Results

### 3.1 Baseline and post demineralization hardness

Mean Hb of sound enamel across all groups ranged from 338 to 393 gf/mm2. After 12 h immersion in acid gel (pH 5.0), mean Hd values decreased considerably after exposure (to approximately 140–141 gf/mm^2^) across all specimens **(**Figure 5**).**

**Figure 5:**
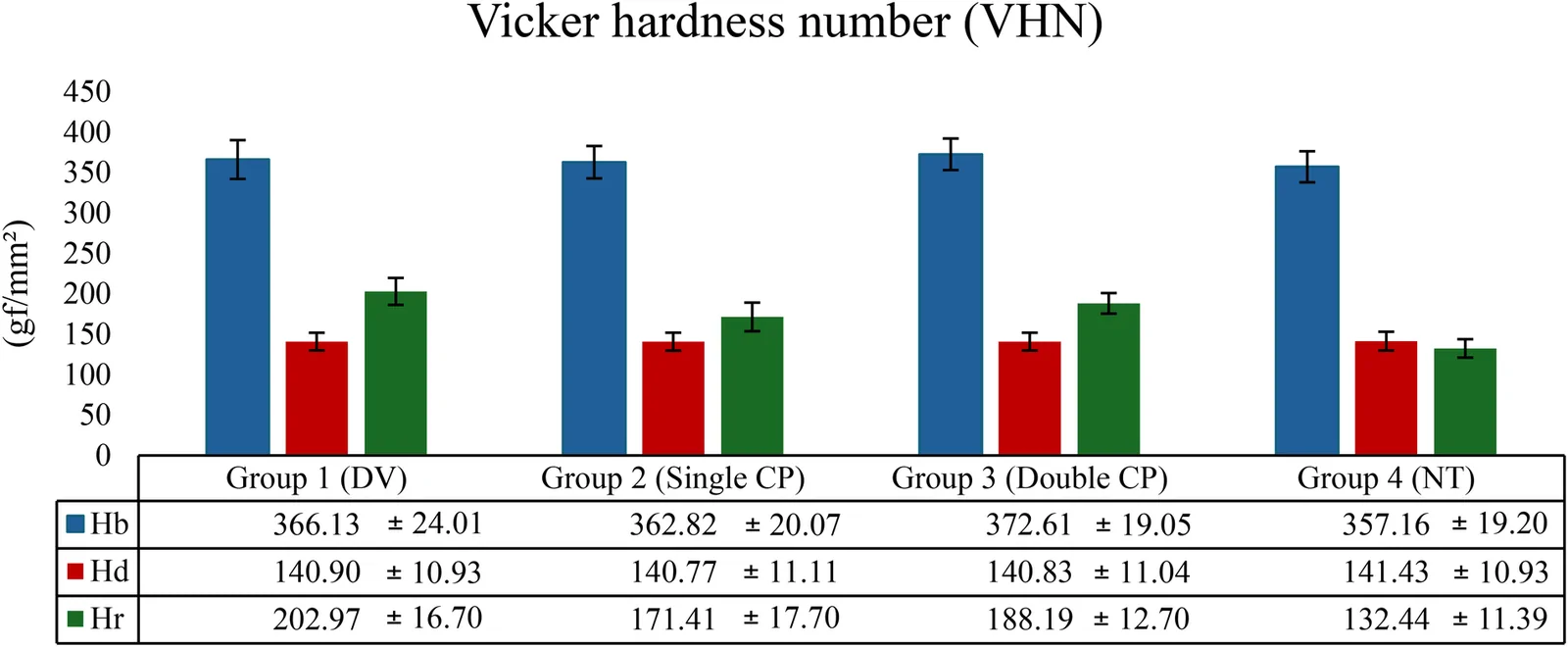
Surface microhardness at different experimental stages: mean and standard deviation of vickers hardness number (baseline hardness; Hb, hardness after demineralization; Hd and hardness after remineralization; Hr).

### 3.2 Remineralized hardness and hardness recovery

After treatment and 7-day pH cycling, mean remineralized hardness (Hr) was highest in Group DV, followed by Group DCP, Group SCP, and Group NT. The NT group showed Hr values lower than Hd, indicating continued net mineral loss in the absence of fluoride treatment.

Welch ANOVA revealed statistically significant differences in hardness recovery (HR = Hr−Hd) among all groups **(**Table 2**)**. Group DV showed significantly greater HR compared with Group SCP (mean difference = 31.37 gf/mm2; *p* < 0.001) and Group NT (mean difference = 71.05 gf/mm2; *p* < 0.001). A statistically significant difference was also found between Group DV and Group DCP (mean difference = 14.71 gf/mm2; *p* = 0.041), and between Group SCP and Group DCP (mean difference = −16.66 gf/mm2; *p* = 0.002).

**Table 2:** *Post hoc* comparisons (games howell) of hardness recovery (HR = Hr−Hd) among groups.

Reference group	Comparison group	Mean Difference (I-J) (gf/mm^2^)	Std. Error	Sig.	95% Confidence Interval	
					Lower Bound	Upper Bound
DV	SCP	31.37059*	5.82967	<0.001	15.5001	47.2411
	DCP	14.70706*	5.13286	0.041	.4537	28.9604
	NT	71.05412*	5.03110	<0.001	57.0093	85.0989
SCP	DV	−31.37059*	5.82967	<0.001	−47.2411	−15.5001
	DCP	−16.66353*	4.02690	0.002	−27.7053	−5.6217
	NT	39.68353*	3.89637	<0.001	28.9390	50.4280
DCP	DV	−14.70706*	5.13286	0.041	−28.9604	−.4537
	SCP	16.66353*	4.02690	0.002	5.6217	27.7053
	NT	56.34706*	2.74642	<0.001	48.8985	63.7956
NT	DV	−71.05412*	5.03110	<0.001	−85.0989	−57.0093
	SCP	−39.68353*	3.89637	<0.001	−50.4280	−28.9390
	DCP	−56.34706*	2.74642	<0.001	−63.7956	−48.8985

* Statistically significant (*p* < 0.05).

Welch ANOVA with Games Howell *post hoc* test. DV, Duraphat™ Varnish; SCP, single Clinpro™ Clear; DCP, double Clinpro™ Clear; NT, non-treatment control.

Both %HR and %RP confirmed these findings **(**Tables 3 and 4**)**. Group DV showed significantly greater %HR compared with Group SCP (*p* < 0.001), Group DCP (*p* = 0.011), and Group NT (*p* < 0.001). Similarly, %RP was significantly higher in DV versus SCP (*p* < 0.001), DCP (*p* = 0.021), and NT (*p* < 0.001). Group DCP showed significantly greater %HR and %RP than Group SCP (*p* = 0.026 and *p* = 0.005, respectively).

**Table 3:** The percentages of hardness recovery (%HR) inter groups.

Reference group	Comparison group	Mean Difference (I-J) (%)	Std. Error	Sig.	95% Confidence Interval	
					Lower Bound	Upper Bound
DV	SCP	13.25588*	2.58506	<0.001	6.2520	20.2597
	DCP	7.00353*	2.04004	0.011	1.3659	12.6412
	NT	31.66706*	2.00270	<0.001	26.1095	37.2247
SCP	DV	−13.25588*	2.58506	<0.001	−20.2597	−6.2520
	DCP	−6.25235*	2.04073	0.026	−11.8920	-.6127
	NT	18.41118*	2.00340	<0.001	12.8515	23.9708
DCP	DV	−7.00353*	2.04004	0.011	−12.6412	−1.3659
	SCP	6.25235*	2.04073	0.026	.6127	11.8920
	NT	24.66353*	1.22182	<0.001	21.3514	27.9757
NT	DV	−31.66706*	2.00270	<0.001	−37.2247	−26.1095
	SCP	−18.41118*	2.00340	<0.001	−23.9708	−12.8515
	DCP	−24.66353*	1.22182	<0.001	−27.9757	−21.3514

* Statistically significant (*p* < 0.05).

Welch ANOVA with Games Howell *post hoc* test. DV, Duraphat™ Varnish; SCP, single Clinpro™ Clear; DCP, double Clinpro™ Clear; NT, non-treatment control.

**Table 4:** The percentages remineralized potential (%RP) inter groups.

Reference group	Comparison group	Mean Difference (I-J) (%)	Std. Error	Sig.	95% Confidence Interval	
					Lower Bound	Upper Bound
DV	SCP	22.97176*	4.06887	<0.001	11.9394	34.0041
	DCP	10.99118*	3.49783	0.021	1.3785	20.6038
	NT	50.77588*	3.58803	<0.001	40.9534	60.5984
SCP	DV	−22.97176*	4.06887	<0.001	−34.0041	−11.9394
	DCP	−11.98059*	3.20562	0.005	−20.7490	−3.2122
	NT	27.80412*	3.30382	<0.001	18.7978	36.8104
DCP	DV	−10.99118*	3.49783	0.021	−20.6038	−1.3785
	SCP	11.98059*	3.20562	0.005	3.2122	20.7490
	NT	39.78471*	2.56793	<0.001	32.8237	46.7457
NT	DV	−50.77588*	3.58803	<0.001	−60.5984	−40.9534
	SCP	−27.80412*	3.30382	<0.001	−36.8104	−18.7978
	DCP	−39.78471*	2.56793	<0.001	−46.7457	−32.8237

* Statistically significant (*p* < 0.05).

Welch ANOVA with Games Howell *post hoc* test. DV, Duraphat™ Varnish; SCP, single Clinpro™ Clear; DCP, double Clinpro™ Clear; NT, non-treatment control.

### 3.3 Polarized light microscope (PLM)

PLM images under 20  ×   magnification by longitudinal ground section confirmed demineralization across all acid-exposed groups **(**Figure 6**)**, evidenced by subsurface dark zones of positive birefringence relative to the healthy control. Group NT displayed a dark band of moderate depth, which was reduced relative to the acid gel control (without pH cycling). All fluoride-treated groups (DV, SCP, and DCP) demonstrated notably smaller dark zones than Group NT, with dark band widths observed across the three groups, indicating broadly similar qualitative reductions in lesion depth regardless of the fluoride agent applied.

**Figure 6:**
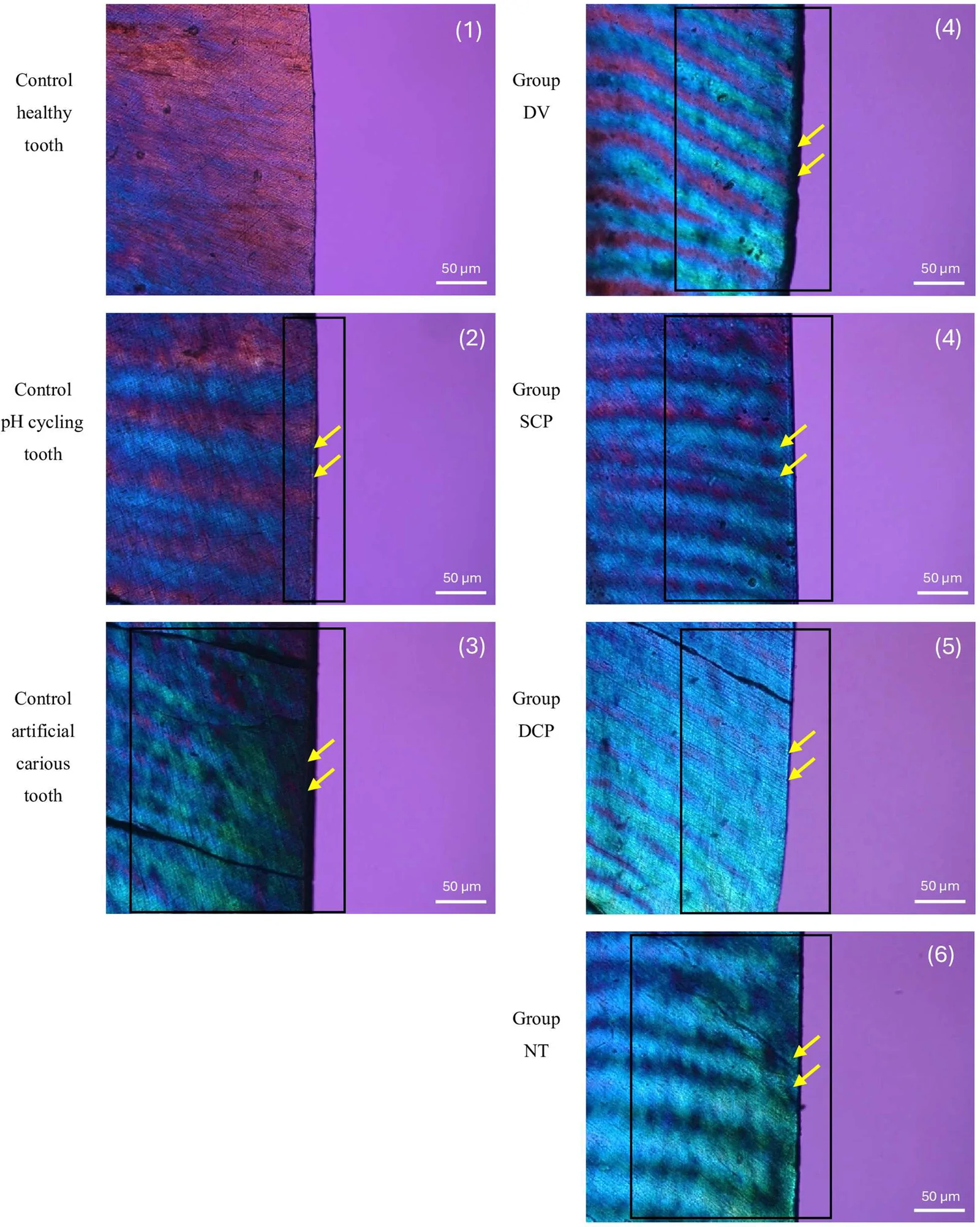
Polarized light microscope findings: PLMimages under 20  ×  magnification of longitudinal ground section; yellow arrows indicate the demineralization area. (1) Control healthy tooth, (2) Control pH cycling tooth, (3) Control artificial carious tooth, (4) Duraphat^TM^ varnish, (5) Single application of Clinpro^TM^Clear Fluoride Treatment, (6) Double application of Clinpro^TM^ Clear Fluoride Treatment, (7) Non-treatment.

### 3.4 Scanning electron microscopy (SEM)

SEM at 2,000  ×   magnification revealed marked surface roughness and structural breakdown in Group NT **(**Figure 7**)**. This was characterized by a distinct honeycomb pattern, reflecting selective dissolution of prism cores with relatively intact prism boundaries, when compared to the acid gel control without pH cycling. All fluoride-treated groups (DV, SCP, and DCP) exhibited greater surface regularity than Group NT, though none fully restored the smooth morphology observed in the healthy control. Notably, Group DV displayed the smoothest surface among the treated groups.

**Figure 7:**
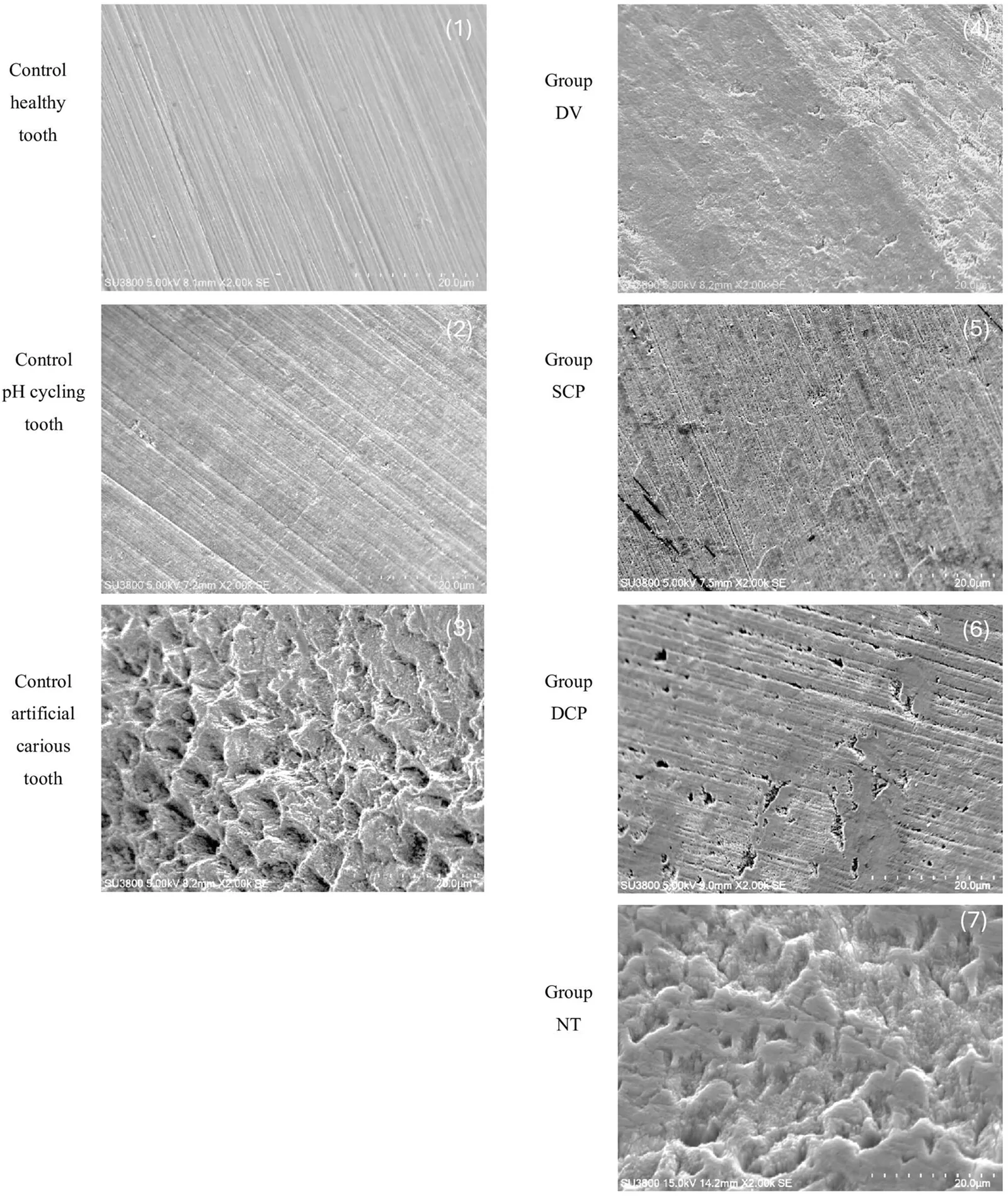
Scanning electron microscopy findings: SEM images under 2,000  ×  magnification; surface roughness and porosity represent the demineralization area. (1) Control healthy tooth, (2) Control pH cycling tooth, (3) Control artificial carious tooth, (4) Duraphat^TM^ varnish, (5) Single application of Clinpro^TM^ Clear Fluoride Treatment, (6) Double application of Clinpr^TM^ Clear Fluoride Treatment, (7) Non-treatment.

### 3.5 Energy dispersive x-ray spectroscopy (EDS)

EDS analysis **(**Table 5**)** revealed distinct differences in elemental composition across the groups. Fluorine was not detected in the untreated group (NT), confirming the absence of fluoride incorporation in this group. In contrast, all three fluoride-treated groups showed measurable fluorine content. Among these groups, Group DV demonstrated the highest fluorine content, followed by Groups DCP and SCP, respectively. Regarding calcium content, all experimental groups showed a reduction compared with the healthy control group, with Group DV showing the smallest degree of reduction. The calcium-to-phosphorus (Ca/P) ratio remained largely similar across all groups.

**Table 5:** Mean elemental composition (weight %) and Ca/P ratio by group as determined by EDS.

Atom	Control healthy tooth		Control pH cycling tooth		Control Artificial carious tooth		Group 1 (DV)		Group 2 (Single CP)		Group 3 (Double CP)		Group 4 (NT)	
	Mean	SD	Mean	SD	Mean	SD	Mean	SD	Mean	SD	Mean	SD	Mean	SD
Calcium (Ca)	33.44	0.12	32.68	1.57	45.37	1.06	27.78	0.59	25.28	1.40	21.04	2.34	27.21	0.90
Phosphorus (P)	15.93	0.01	15.65	0.49	18.39	0.16	12.93	0.32	12.42	0.67	12.98	1.14	12.99	0.63
Fluorine (F)	ND	ND	ND	ND	ND	ND	0.80	0.29	0.28	0.21	0.46	0.25	ND	ND
Oxygen (O)	40.04	0.15	37.03	2.50	24.15	1.12	36.35	0.37	35.83	4.4	38.42	1.73	35.32	4.03
Carbon (C)	9.51	0.54	13.57	1.06	11.18	0.48	21.34	0.91	25.03	6.33	20.33	2.10	23.78	4.83
Ca/P ratio	2.17	0.01	2.15	0.03	2.50	0.04	2.21	0.03	2.13	0.02	2.15	0.02	2.15	0.06

ND, not detected.

Control healthy tooth; Control pH cycling tooth; Control artificial carious tooth; DV, Duraphat™ Varnish; SCP, single Clinpro™ Clear; DCP, double Clinpro™ Clear; NT, non-treatment.

## 4 Discussion

The present study provides the first direct comparison of Clinpro™ Clear Fluoride Treatment with Duraphat™ Varnish on artificially induced WSLs around orthodontic brackets. On the basis of these findings, the null hypothesis is rejected. The results confirm that Duraphat™ Varnish remains the superior fluoride agent for WSLs remineralization. Notably, however, double application of Clinpro™ Clear produced a meaningful improvement over single application.

 (30, 32–35)The superior hardness recovery observed in the group DV likely reflects two main factors: fluoride concentration and material viscosity. Duraphat™ is a rosin-based sodium fluoride varnish containing 22,600 ppm fluoride, whereas Clinpro™ Clear is a water-based sodium fluoride treatment containing 9,500 ppm fluoride. These differences in formulation may influence fluoride release kinetics, material viscosity, and retention on the enamel surface, thereby affecting the duration of fluoride availability for remineralization. The thick consistency of Duraphat™ also promotes longer retention on the enamel surface, which is consistent with previous reports (30, 32–35). A rosin-based varnish generally forms a hydrophobic film that may remain on the tooth surface for a longer period, potentially serving as a fluoride reservoir and providing sustained fluoride release. In contrast, water-based formulations such as Clinpro™ Clear may exhibit different dissolution characteristics and fluoride release profiles. Nevertheless, these proposed mechanisms, including fluoride release kinetics, material viscosity, and enamel retention time, were not directly evaluated in the present study and should therefore be regarded as possible explanations rather than definitive causes of the observed differences between the two formulations.

The finding that Group DCP performed significantly better than Group SCP in both percentage hardness recovery (%HR) and percentage remineralization potential (%RP) (*p* < 0.05) supports the notion that increased application frequency can compensate for the lower fluoride concentration of Clinpro™ Clear Fluoride Treatment in this study. Further research may explore whether this offers a suitable alternative for patients who find the appearance of conventional fluoride varnish aesthetically undesirable.

PLM provided qualitative support for the microhardness findings. All fluoride-treated groups demonstrated a reduction in dark band compared with the untreated control (NT). As a cost-effective qualitative technique, PLM allows for the identification of microstructural characterization in WSLs, thereby complementing and reinforcing the results obtained from surface microhardness testing (30, 32, 36–41). Group NT displayed a dark band of moderate depth, which was reduced compared to the acid gel control (without pH cycling), reflecting partial remineralization from the remineralizing solution from pH cycling. The comparable dark zone sizes observed across Groups DV, SCP, and DCP likely reflect the inherent limitations of PLM in detecting subtle intergroup differences, indicating broadly similar qualitative reductions in lesion depth regardless of the fluoride agent applied. Notably, in Group DV, the varnish covered the enamel surface, as evidenced by the presence of a thin, distinct dark line observed along the enamel surface.

SEM analysis revealed notable differences in surface morphology across the groups. The healthy control group exhibited a smooth and uniform enamel surface with no visible signs of porosity, serving as a reference for intact enamel structure. In contrast, all fluoride-treated groups displayed greater surface irregularity, which may be attributed to two factors. First, the pH cycling process itself may have induced mild surface roughness and slight porosity as a result of the alternating demineralization and remineralization cycles, consistent with a previously reported finding (42). Second, incomplete fluoride-induced remineralization following repeated acid challenges may have contributed to the residual surface changes observed.

Among the fluoride-treated groups, Group DV demonstrated the most effective remineralization, as evidenced by a relatively smooth and uniform enamel surface. However, the thick consistency of the varnish made complete removal more difficult, resulting in irregular particle deposits remaining on the enamel surface, a finding also noted in a previous study (43). Groups SCP and DCP, which utilized water-based gel formulations, showed remineralization outcomes broadly comparable to those of Group DV. Nevertheless, residual surface damage remained visible in these groups, likely due to their lower viscosity and reduced retention on the enamel surface during pH cycling, which may have diminished their overall protective effect. Group NT exhibited considerable surface roughness and porosity, second only to the acid gel control group, which displayed the most severe structural damage, characterized by a highly irregular surface and numerous pores resulting from prolonged acid exposure (44). All fluoride-treated groups (DV, SCP, and DCP) suggested superior surface remineralization relative to the non-treatment group.

EDS is a valuable tool in studies of fluoride-induced enamel remineralization, as it enables direct measurement of changes in elemental composition at the tooth surface, particularly calcium, phosphorus, and fluorine, before and after treatment. This allows researchers to determine whether fluoride therapy is genuinely restoring mineral content rather than merely improving surface appearance (45). In the present study, EDS results demonstrated a measurable increase in fluorine content at the enamel surface across all treated groups. The higher fluorine content observed in Group DV is consistent with the mechanism described above, namely greater fluoride concentration and prolonged surface contact. Comparable fluorine levels were observed in Groups DCP and SCP, reflecting the similar fluoride concentrations present in both water-based formulations. These findings are in agreement with those reported by Fathollah et al., who demonstrated a significant increase in fluoride atomic percentage from 1.21% to 7.31% at 48 h following fluoride varnish application, accompanied by an elevated calcium-to-phosphorus (Ca/P) ratio, as confirmed by SEM-EDX analysis (46).

Notably, Ca and P weight percentages in the present study remained relatively stable after treatment. This finding is supported by Vicente et al., who reported stable Ca and P percentages alongside detectable fluoride in the fluoride varnish treated group (47). The semi-quantitative nature of EDS limits analysis to the surface layer, and additional techniques such as micro CT or Raman spectroscopy may provide deeper compositional data in future studies.

Three main *in vitro* models have been developed to simulate the caries process: pH cycling models, biofilm-based microbiological models, and simple immersion models. pH cycling models most closely replicate the alternating demineralization and remineralization cycles of the oral environment by using acidic buffers at pH 4.0 to 5.0 in combination with remineralizing solutions (48). Microbiological models employ caries-associated bacteria, such as Streptococcus mutans, to simulate biofilm-mediated caries progression, offering greater clinical relevance but lower reproducibility. Simple immersion models, while less biologically realistic (49), provide experimental simplicity and consistency, making them well suited for controlled lesion formation studies (50).

In the present study, a simple immersion gel-based demineralizing model using lactic acid was employed. Within immersion-based approaches, gel-based models incorporate thickening agents that more closely simulate the stagnant plaque layer on the tooth surface, producing lesions that are more uniform and natural in appearance compared with those generated by liquid solution models (51). Although this model offers less clinical relevance than microbiological models in terms of replicating the natural development of WSLs, its key advantage lies in producing reproducible and measurable demineralization, which is consistent with the objectives of the present study.

The pH cycling approach is widely accepted as a laboratory method that simulates the natural alternation between demineralization and remineralization in the oral environment (52). Although *in vitro* pH cycling data may not fully replicate *in situ* or clinical conditions, surface microhardness recovery measurements have been shown to provide a reasonable estimate of remineralization outcomes observed *in situ* (53). DI water was used throughout pH cycling, in place of artificial saliva, to eliminate confounding mineral ions and buffering components and to isolate the effect of the tested fluoride treatments. A 7-day cycling duration was selected based on prior studies demonstrating that this period is sufficient to produce measurable mineral changes in enamel while remaining experimentally practical (54, 55). This duration is also consistent with reports supporting the use of a 7-day pH-cycling protocol for evaluating enamel changes in artificial white spot lesions (23, 56–58). The remineralization observed following fluoride treatment aligns with the established mechanism of fluoride action, namely the promotion of fluorapatite formation and the reduction of mineral dissolution during acid challenge (59). This finding confirms that the model was capable of detecting meaningful fluoride uptake and enamel recovery. Although a longer cycling period might theoretically produce greater lesion depth or more distinct remineralization differences, evidence indicates that meaningful differences between treatment groups can be reliably detected within 6–10 days under standardized cycling conditions (60, 61).

This study has several limitations inherent to *in vitro* research. The pH-cycling model cannot fully replicate the complexity of the oral environment, including the effects of saliva buffering, protein pellicle formation, microbial biofilms, and patient behavior. The use of DI water rather than artificial saliva may have led to an underestimation of the remineralizing potential across all groups (62, 63). Additionally, micro-CT analysis represents a more advanced approach that could more accurately capture remineralization depth and surface changes; however, it remains costly and not widely accessible. Finally, these results should be confirmed through *in situ* or clinical trials before definitive clinical recommendations can be made.

The present findings may have potential clinical implications for the management of WSLs during orthodontic treatment. Although Duraphat™ varnish demonstrated the greatest remineralization potential under the present *in vitro* conditions, Clinpro™ Clear Fluoride Treatment also promoted enamel remineralization, particularly when applied twice. This finding suggests that application frequency may influence the remineralization efficacy of lower-fluoride formulations. Therefore, in clinical practice, the selection of a fluoride agent should not rely solely on fluoride concentration but should also consider treatment protocols, patient compliance, and clinical circumstances.

From a practical perspective, Duraphat™ varnish is generally applied as a professional topical varnish that adheres firmly to the enamel surface and may require less frequent application because of its prolonged retention. In contrast, Clinpro™ Clear Fluoride Treatment is a water-based professional fluoride treatment with a lower fluoride concentration and may require repeated applications to achieve comparable remineralization outcomes. The shorter application time, transparent appearance, and ease of removal may improve patient acceptance, particularly in orthodontic patients concerned about aesthetics. Furthermore, the frequency of professional application, chairside time, and overall cost-effectiveness should be considered when selecting an appropriate fluoride treatment in routine clinical practice.

Finally, although the present *in vitro* model provides a standardized environment for comparing fluoride formulations, clinical performance may also be influenced by saliva, dental biofilm, dietary habits, oral hygiene practices, and patient compliance. Consequently, well-designed *in vivo* and randomized clinical studies are warranted to determine whether the superior remineralization observed under laboratory conditions can be translated into long-term clinical benefits.

## 5 Conclusion

Notably, the double application of Clinpro™ Clear Fluoride Treatment significantly improved its remineralization efficacy compared with the single application and untreated control groups, suggesting that increased application frequency may partially compensate for its lower fluoride concentration. Further *in vivo* studies are warranted to confirm these findings under clinical conditions.

## Data Availability

The original contributions presented in the study are included in the article/Supplementary Material, further inquiries can be directed to the corresponding author.
